# Characteristics of Gorilla-Specific *Lactobacillus* Isolated from Captive and Wild Gorillas

**DOI:** 10.3390/microorganisms6030086

**Published:** 2018-08-14

**Authors:** Sayaka Tsuchida, Steven Kakooza, Pierre Philippe Mbehang Nguema, Eddie M. Wampande, Kazunari Ushida

**Affiliations:** 1 Academy of Emerging Sciences, Chubu University, 1200 Matsumoto-cho, Kasugai-shi, Aichi 487-8501, Japan; s_tsuchida@isc.chubu.ac.jp; 2 Graduate School of Life and Environmental Sciences, Kyoto Prefectural University, Shimogamo, Kyoto 606-8522, Japan; 3 Central Diagnostic Laboratory, College of Veterinary Medicine, Animal Resources and Biosecurity, Makerere University, P.O. Box 7062 Kampala, Uganda; ksteven310@gmail.com (S.K.); ewampande@yahoo.co.uk (E.M.W.); 4 Novel Minds Science Plexus, P.O. Box 7062 Kampala, Uganda; 5 Research Institute of Tropical Ecology, BP 13354 Libreville, Gabon; mbehangnguema@yahoo.fr

**Keywords:** *Lactobacillus gorillae*, western lowland gorilla, mountain gorilla, phylogeny, phenotypic characteristic, antipathogenic infection

## Abstract

Lactic acid bacteria (LAB) reside in a wide range of mammals, such as autochthonous intestinal bacteria. In this paper, we present the phenotypic and phylogenetic characteristics of gorilla-specific LAB. *Lactobacillus gorillae*—previously isolated from the wild and captive western lowland gorillas (*Gorilla gorilla gorilla*)—were successfully isolated from wild mountain gorillas (*Gorilla gorilla beringei*) in addition to other captive and wild western lowland gorillas. The strains from wild gorillas could ferment D-xylose, arbutine, cellobiose, and trehalose better than those from captive gorillas. By contrast, tolerance to NaCl was higher in isolates from captive gorillas than in those from wild gorillas. This tendency may have been induced by regular foods in zoos, which contain sufficient amount of salts but less amount of indigestible fiber and plant secondary metabolites compared to foods in the wild. All strains of *L. gorillae* showed inhibitory activities to enteric pathogenic bacteria; however, the activity was significantly higher for strains from wild gorillas than for those from captive gorillas. This may have been induced by the captive condition with routine veterinary intervention. Since *L. gorillae* can grow in the gastrointestinal tract of gorillas in captivity, the strains from wild mountain gorillas are potential probiotics for gorillas under captive conditions.

## 1. Introduction

The gastrointestinal tract (GIT) microbes of mammals develop complex ecosystems with a vast diversity after birth, which largely varies between animal species and individuals [1,2]. Accordingly, host species, particularly humans, are now recognized as being made up of superorganisms together with indigenous microbes [2,3,4]. Lactic acid bacteria (LAB), which are narrowly defined as bifidobacteria and lactobacilli, have been especially well studied because of their beneficial functions in humans. Many studies are concerned with the beneficial functions of LAB, for example, the immune-stimulating and modulation effect caused by the interaction between LAB and the intestinal mucosa of the host and its role in elevating the host’s defense against pathogenic penetration [5,6,7,8,9]. In addition, LAB are common members of the GIT microbes in a wide range of animals [10], and each animal species hosts autochthonous species of LAB in their GIT [11,12]. Some reports have described an essential role of diet in the development and maintenance of LAB in various animal hosts in captivity [11,13]; however, only few studies have revealed the composition of LAB in wild animals under wild feeding conditions. Specifically, the eating habits of herbivores and omnivores in the wild are quite different from those in captive conditions. Foods available in the wild habitats, particularly for herbivores, deploy several protective chemicals to escape from herbivory [14]. These plant secondary metabolites, particularly toxins, may negatively affect GIT microbes including LAB [15,16], but some GIT microbes can degrade these compounds to help the host to survive on such natural foods in the wild [17,18]. In our previous study, we demonstrated the efficient degradation of plant secondary metabolites and toxins by cecal bacteria in wild Japanese Rock ptarmigans (*Lagopus muta japonica*) [19,20].

In the present study, we have focused on *Lactobacillus gorillae* as a host-specific LAB, which has previously been proposed as a novel species of the genus *Lactobacillus* [21] and is considered as autochthonous LAB of gorillas. We have found that the phylogenetic relationship among strains of *L. gorillae*—isolated from various sources, such as captive and wild western lowland gorillas (*Gorilla gorilla gorilla*) and wild mountain gorillas (*Gorilla gorilla beringei*)—show the monophyly of isolates from *G.g.b*. isolates, apparently separated from those from captive and wild *G.g.g*. We have also shown the phenotypic characteristics and antimicrobial activities of various *L. gorillae* isolates and have demonstrated marked phenotypic differences between wild and captive *G.g.g*.

## 2. Materials and Methods

### 2.1. Bacterial Strains and Growth

The strains of *L. gorillae* used in the present study are shown in Table 1. Strains KZ01^T^, KZ02, KZ03, and GG02 were previously isolated from captive and wild western lowland gorillas [21]. Strains UM01, UM03, UR07, UR10, and UH14 were isolated from wild mountain gorillas in Bwindi Impenetrable National Park, Uganda; strains HZ04, HZ07, HZ10, HZ11, and HZ16 were isolated from captive western lowland gorillas in Higashiyama Zoo, Nagoya, Japan; and strains GG05, GG08, GG12, and GG15 were isolated from wild western lowland gorillas in Moukalaba-Doudou National Park, Gabon using a method described previously [22]. *L. fermentum* JCM 1173^T^, which is the closest neighbor to *L. gorillae*, was used as the control strain. Bacterial cultures were anaerobically grown on Lactobacilli MRS agar (BD Difco, Fisher Scientific, Ottawa, ON, Canada) at 37 °C for 48 h and stored at −80 °C as stock cultures for further analyses.

### 2.2. Phylogenic Analysis of 16S rRNA Gene

The partial 16S rRNA gene sequences were amplified as described previously [23]. These amplicons were sequenced at Hokkaido System Science Co., Ltd. (Sapporo, Japan) by dye-terminator method using ABI PRISM 3100 Genetic Analyzer (Applied Biosystems). Calculation of pairwise 16S rRNA gene sequence similarities was performed using MEGA version 7.0.20 [24]. Multiple sequence alignments were performed using the CLUSTAL W program [25], and phylogenetic trees were constructed using the neighbor-joining method [26] with Kimura’s two-parameter model [27] using MEGA version 7.0.20. Tree topology was evaluated by a bootstrap analysis with 1000 replicates using CLUSTAL W.

### 2.3. Phenotypic Characteristics

Biochemical characteristics were evaluated using API 50 CH and ZYM systems (bioMerieux, Marcy-l’Étoile, France) according to the manufacturer’s instructions. Tolerance to NaCl was examined in anaerobic MRS broth containing 6.5, 8.0, and 10% (*w*/*v*) NaCl. The positive or negative growth was determined by turbidity after 7 days at 37 °C incubation.

### 2.4. Antimicrobial Activity Assay

The agar well diffusion method as described by Onda et al. [28] was used with some minor modifications. In brief, the suspension of target enterohemorrhogic *Escherichia coli* (EHEC) adjusted to McFarland standard NO. 0.5 was inoculated on Muller Hinton agar (BD Difco) plates, and well holes were punched out of the agar using a cork borer. The wells were then filled with 75 µL of MRS culture supernatant of *L. gorillae* (adjusted to pH 7.0 by 0.1 N NaOH). The agar plates were aerobically incubated at 37 °C for 18 h. After incubation, the diameters (mm) of the inhibition zone were measured as antimicrobial activities. Comparison was made using Welch’s test in R (version 3.1.2) for the diameter of inhibition zone between strains from mountain gorillas in the wild (MW), those from western lowland gorillas in captivity (WC), and those from western lowland gorillas in the wild (WW).

The presently used EHEC (Strain Ehime) was kindly provided by Dr. Nobuo Nakanishi, KYODOKEN Institute (Fushimi, Kyoto 612-8073 Japan). The beta hemorrhagic strain was previously isolated from a piglet in a commercial pig farm in Ehime Prefecture, Japan, and characterized as Stx2e(+), AIDA1(+), and F18(+).

### 2.5. Antibiotic Resistance Profile

The 18 strains of *L. gorillae* were examined for their susceptibility to imipenem, cefotaxime, ofloxacin, amoxicillin, gentamicin, lincomycin, tetracycline, and erythromycin by the disk diffusion test.

The cells were grown on MRS agar under anaerobiosis at 37 °C for 18 h. A portion of colony was taken and suspended in Muller Hinton broth to McFarland standard NO. 0.5. Imipenem (10 µg/disk), cefotaxime (30 µg/disk), ofloxacin (5 µg/disk), amoxicillin (25 µg/disk), gentamicin (10 µg/disk), lincomycin (2 µg/disk), tetracycline (30 µg/disk), and erythromycin (15 µg/disk) disks were obtained from Eiken Chemical Co., Ltd. (Tochigi, Japan) and Nissui Pharmaceutical Co., Ltd. (Tokyo, Japan), and applied according to the manufacturer’s instructions. The inhibition zone was determined after 18 h of culture.

### 2.6. Ethics

Sampling of feces from gorillas was realized in a noninvasive manner under the supervision of either rangers, officers of the National Park, or officers of National Institutes in Uganda and Gabon. The research in Uganda was approved both by Uganda National Council for Science & Technology (UNCST) as NS540 (11 September 2015) and by Uganda Wildlife Authority (UWA) as EDO35-01 (11 August 2015). Research in Gabon was approved by Centre National de la Recherche Scientifique et Technique (CENAREST) as AR0033/11 (9 November 2011). Feces from captive gorillas were supplied by officers of the Kyoto City Zoo and the Higashiyama Zoo under the contract established between Kyoto Prefectural University and the director of both zoos.

## 3. Results

### 3.1. Phylogenetic Analysis of 16S rRNA Gene

**Figure 1:**
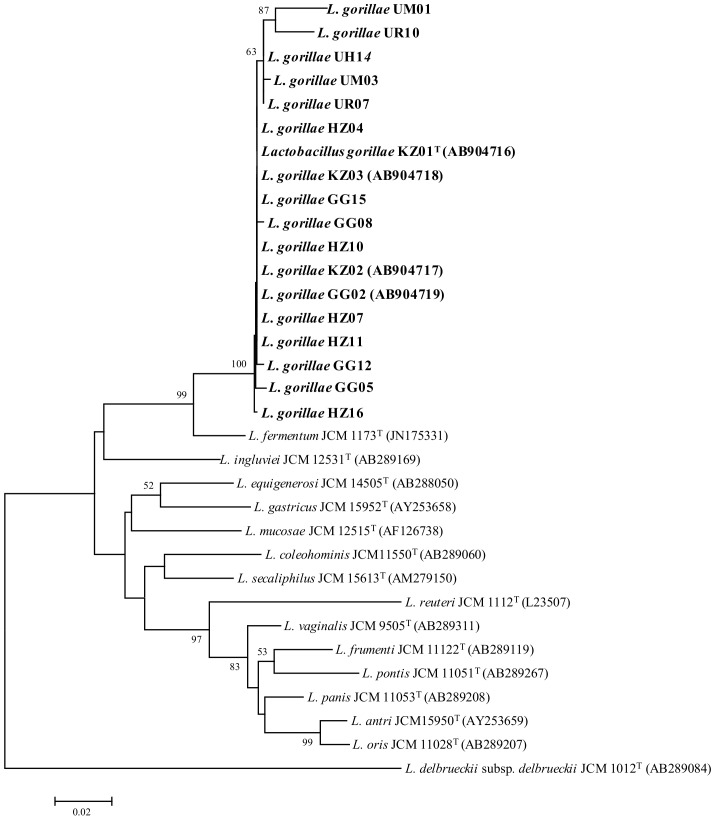
Phylogenetic tree based on partial 16S rRNA gene sequences showing the relationships between *L. gorillae* and members of related *Lactobacillus* species. The tree was conducted by neighbor-joining method. *L. delbruekii* subsp. *delbrueckii* JCM 1012^T^ was used as an out-group. Bootstrap values (>50%) based on 1000 replicates are shown at branch nodes. Bars represent 0.02 substitutions per nucleotide position.

According to 16S rRNA gene sequence phylogeny, strains of *L. gorillae* from western gorillas formed different monophyletic cluster from that of strains from mountain gorillas (Figure 1). The sequences of strains UR07 and UH14 showed the highest similarities to those of strain KZ01^T^, which is a type strain of *L. gorillae*, (99.8%) and to those of strain UM01, which presented with the lowest similarity to those of strain KZ01^T^ (97.6%).

### 3.2. Phenotypic Characteristics

According to the API 50 CH and API ZYM results, all strains of *L. gorillae* possessed similar phenotypic traits as those of *L. fermenum* JCM 1173^T^, except for naphthol-AS-BI-phosphohydrolase, α-galactosidase, and α-glucosidase. Acid production from D-xylose, arbutin, esclin, salicin, cellobiose, lactose, trehalose, gluconate, and 2-keto-gulconate showed strain variations among the 18 strains of *L. gorillae*. In particular, acid was produced from 2-keto-gulconate by only the strains from mountain gorillas. For the enzyme activity pattern, cystine arylamidase was detected in only the strains from mountain gorillas (Table 1). None of the strains of *L. gorillae* could grow in MRS broth with the presence of 10% (*w*/*v*) NaCl, whereas all strains grew in the presence of 6.5% (*w*/*v*) NaCl. Only the strains from captive individuals (KZ01^T^, KZ02, and KZ03) grew in the presence of 8% (*w*/*v*) NaCl (Table 2).

### 3.3. Antimicrobial Activity Assay

**Figure 2:**
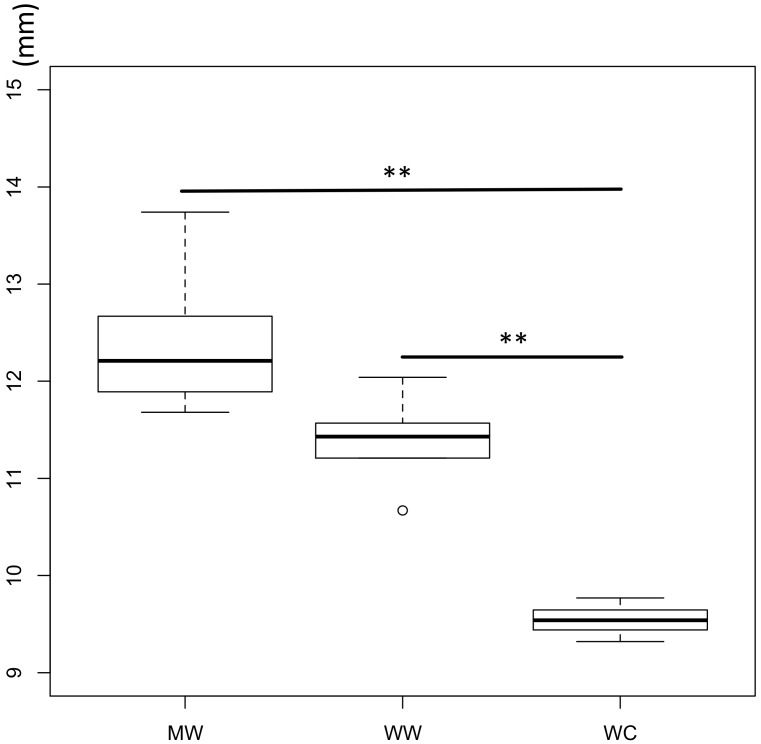
Antimicrobial activities of strains from gorillas. MW—strains from wild mountain gorillas (*n* = 5); WW—strains from wild western lowland gorillas (*n* = 5); WC—strains from captive western lowland gorillas (*n* = 8). In the box plot, the line in the middle of the box represents the median; the upper and lower perimeters of the box represents the 75th and 25th centiles, respectively; and the tails represent 2.5th and 97.5th centiles, respectively. ** *p* < 0.01.

All strains of *L. gorillae* inhibited the growth of EHEC to some extent. Antimicrobial activities determined as the inhibitory zone (mm) of all strains are shown in Figure 2. The inhibitory activities of strains from wild mountain gorillas (MW), wild western lowland gorillas (WW), and captive western lowland gorillas (WC) ranged from 11.68 to 13.74, 10.64 to 12.4, and 9.32 to 9.77, respectively. The inhibitory activities to EHEC were significantly higher for MW and WW than for those of WC (*p* < 0.01). However, no significant difference was observed in the inhibitory activities to EHEC between MW and WW (*p* = 0.072).

### 3.4. Antibiotic Resistance Profile

The antibiotic resistance profiles of *L. gorillae* are shown in Table 3. The disk diffusion test confirmed the resistance to cefotaxime for strains KZ02 and HZ11 from captive western lowland gorillas. Strains from captive western lowland gorillas KZ01^T^, HZ04, HZ07, and HZ10 also showed intermediate resistance to cefotaxime. Resistance to ofloxacin was confirmed in all strains from captive western lowland gorillas and wild mountain gorillas. None of strains tested showed resistance or intermediate resistance to imipenem, amoxicillin, gentamicin, lincomycin, tetracycline, and erythromycin according to the disk diffusion test.

## 4. Discussion

In the present study, we successfully isolated *L. gorillae* from the feces of captive and wild western lowland gorillas and wild mountain gorillas in addition to previously isolated *L. gorillae*. Since *L. gorillae* has been repeatedly isolated from western lowland and mountain gorilla, *L. gorillae* can be regarded as an autochthonous GIT microbe of the genus *Gorilla*. Interestingly, the phylogenetic analysis of 16S rRNA gene sequences revealed that *L. gorillae* formed two distinct phylogenetic clusters comprising either strains from western lowland gorillas or those from mountain gorillas (Figure 1). We hypothesized the divergence time of these two clusters of *L. gorillae* as 1.75 million years ago because the divergence time of western and eastern gorilla was estimated as 1.75 million years ago by whole genomic study of gorilla [29]. Based on our hypothesis on the divergence time of the two *L. gorillae* clusters, the divergence time of human-associated *Lactobacillus* (*L. fermentum*) and gorilla-specific *Lactobacillus* (*L. gorillae*) was calculated to be 8.3 million years old (Figure S1). *L. fermentum* is the phylogenetic neighbor of *L. gorillae* and can be regarded as human-associated LAB [30]. This divergence time for gorilla-specific LAB and its phylogenetic neighbor—a human-associated LAB—seems reasonable because the common ancestor of gorillas and humans existed until some 7–10 million years ago in the forest of Africa [29,31,32]. Thus, our hypothesis about the divergent time of 1.75 million years is plausible as the divergent time between the two groups of *L. gorillae*. Despite such an ancient phylogenetic separation, the strains from wild western lowland gorillas showed virtually the same phenotypic characteristics with those from wild mountain gorillas. A difference in 2-keto-gluconate utilization, the presence of cysteine arylamidase, and 66 other characteristics were found to be the same. However, the difference in phenotypic characteristics became more visible when the strains from wild gorillas were compared with those from captive gorillas. In fact, strains from wild gorillas, both western lowland and mountain gorillas, could utilize different types of sugars compared with strains from captive gorillas (Table 2). Considering the food composition of wild gorillas, the fermentation of D-xylose, cellobiose, and arbutin by gorilla-specific GIT microbes is important. D-xylose and cellobiose constitute hemicelluloses and cellulose of the plant cell wall, which are obviously rich in gorilla’s natural food items. Moreover, arbutin is a phenolic glycoside contained in wild fruits and leaves, which helps to avoid animal herbivory with its cytotoxicity [33]. The degradation of these plant secondary metabolites is an essential functionality of GIT bacteria in wild herbivore animals toward supporting the host-feeding behavior [17,19,20,34].

In addition to sugar utilization, tolerance to NaCl also showed interesting traits. All eight strains from captive gorillas showed higher tolerance to NaCl than those from wild gorillas. The food items in zoos contained sufficient, or sometimes excessive, amount of salts, whereas minerals, particularly sodium, were often depleted from the natural food items of wild gorillas. In fact, wild western lowland gorillas often consume unusual food items that are relatively rich in sodium, such as decayed wood bark or particular water plants [35,36]. Therefore, these differences in phenotypic characteristics between strains from wild and captive gorillas may have developed quite recently because the successful artificial feeding to the gorillas was recorded only in the mid-20th century. In fact, the first gorilla born under captivity was recorded in 1956 in USA. Selection by food items in captivity may have induced these phenotypic modifications. We have obtained the draft genome sequence of *L. gorillae* KZ 01^T^ previously [37]. Since KZ 01^T^ strain originated from a captive western lowland gorilla, further studies are needed to compare it with strains from wild gorillas in order to understand the genetic background of these phenotypic differences in the strains of *L. gorillae*.

According to the result of antimicrobial activity assay, all strains possessed the inhibitory activities to EHEC, and this activity was significantly higher for strains from wild gorillas than for those from captive gorillas (Figure 2). Therefore, it is suggested that *L. gorillae* produces some kind of bactericidal or bacteriostatic substances against enteric pathogenic bacteria, and strains from wild gorillas seem to be more health-promoting in terms of the prevention of enteric pathogenic infection. Veterinary intervention with drugs may have reduced the preventive effect of *L. gorillae* in captivity. Since the strains from wild mountain gorillas showed the highest activity, *L. gorillae* from wild mountain gorillas can be considered as the most beneficial strain for the health promotion of gorilla. Because *L. gorillae* can grow in the GIT of gorillas in captivity, the strains from wild mountain gorillas are considered good candidates for the probiotics under captive conditions. However, it was unexpected that the strains from mountain gorillas showed the same level of resistance to ofloxacin as the strains from the captive gorillas exhibited. Since the transferable antibiotic resistance in probiotic strains has become a serious safety concern [38], it is still advisable that analysis is carried out to understand the precise mechanisms of the drug resistance before direct feeding of these isolates is carried out on captive gorillas.

## Figures and Tables

**Table 1:** The strains of *L. gorillae* used in this study.

Strain ID	Animal Species	Captive or Wild
KZ01^T^ (JCM 19575^T^)	Western lowland gorilla	Captive
KZ02 (JCM 19576)	Western lowland gorilla	Captive
KZ03 (JCM 19577)	Western lowland gorilla	Captive
HZ04	Western lowland gorilla	Captive
HZ07	Western lowland gorilla	Captive
HZ10	Western lowland gorilla	Captive
HZ11	Western lowland gorilla	Captive
HZ16	Western lowland gorilla	Captive
GG02 (JCM 19574)	Western lowland gorilla	Wild
GG05	Western lowland gorilla	Wild
GG08	Western lowland gorilla	Wild
GG12	Western lowland gorilla	Wild
GG15	Western lowland gorilla	Wild
UM01	Mountain gorilla	Wild
UM03	Mountain gorilla	Wild
UR07	Mountain gorilla	Wild
UR10	Mountain gorilla	Wild
UH14	Mountain gorilla	Wild

**Table 2:** Differential phenotypic characteristics of the strains of *L. gorillae*.

Animal Species	Western Lowland Gorilla													Mountain Gorilla					Prevalent in Human
Isolated from	Captive Individuals								Wild Individuals										Fermented Food
Characteristic	KZ01^T^	KZ02	KZ03	HZ04	HZ07	HZ10	HZ11	HZ16	GG02	GG05	GG08	GG12	GG15	UM01	UM03	UR07	UR10	UH14	*L. fermentum* JCM 1173^T^
Acid production from (API 50CH):																			
D-xylose	−	−	+	−	−	+	+	+	+	+	+	+	+	+	+	+	+	+	−
Arbutin	−	−	w	−	−	w	−	+	w	w	w	w	w	+	w	w	+	w	−
Esculin	+	−	+	+	+	+	+	+	+	+	+	+	+	w	+	+	+	w	−
Salicin	−	−	−	−	−	−	−	−	w	w	w	w	w	+	+	w	+	+	−
Cellobiose	−	−	w	−	w	w	-	+	w	w	w	w	w	+	+	+	+	+	−
Lactose	−	−	+	−	−	+	-	+	w	w	w	+	w	+	+	+	+	+	+
Trehalose	−	−	+	−	−	+	-	+	+	+	+	+	+	+	+	+	+	+	−
Gluconate	−	−	+	−	−	w	-	w	w	w	w	w	+	+	+	+	+	+	w
2-keto-gluconate	−	−	−	−	−	-	-	-	-	-	-	-	-	+	+	+	+	+	−
API ZYM results:																			
Cystine arylamidase	−	−	−	−	−	−	−	−	−	−	−	−	−	+	+	+	+	+	+
Naphthol-AS-BI-phosphohydrolase	−	−	−	−	−	−	−	−	−	−	−	−	−	−	−	−	−	−	+
α-galactosidase	+	+	+	+	+	+	+	+	+	+	+	+	+	+	+	+	+	+	−
α-glucosidase	−	−	−	−	−	−	−	−	−	−	−	−	−	−	−	−	−	−	+
Growth in Nacl																			
6.5%	+	+	+	+	+	+	+	+	+	+	+	+	+	+	+	+	+	+	−
8%	+	+	+	+	+	+	+	+	−	−	−	−	−	−	−	−	−	−	−

+: positive, −: negative, W: weak.

**Table 3:** Antibiotic resistant profile of the strains of *L. gorillae*.

Animal Species	Western Lowland Gorilla													Mountain Gorilla				
Isolated from	Captive Individuals								Wild Individuals									
Characteristic	KZ01^T^	KZ02	KZ03	HZ04	HZ07	HZ10	HZ11	HZ16	GG02	GG05	GG08	GG12	GG15	UM01	UM03	UR07	UR10	UH14
Antibiotics:																		
Imipenem (10 µg/disk)	S	S	S	S	S	S	S	S	S	S	S	S	S	S	S	S	S	S
Cefotaxime (30 µg/disk)	I	R	S	I	I	I	R	S	S	S	S	S	S	S	S	S	S	S
Ofloxacin (5 µg/disk)	R	R	R	R	R	R	R	R	S	S	S	S	S	R	R	R	R	R
Amoxicillin (25 µg/disk)	S	S	S	S	S	S	S	S	S	S	S	S	S	S	S	S	S	S
Gentamicin (10 µg/disk)	S	S	S	S	S	S	S	S	S	S	S	S	S	S	S	S	S	S
Lincomycin (2 µg/disk)	S	S	S	S	S	S	S	S	S	S	S	S	S	S	S	S	S	S
Tetracycline (30 µg/disk)	S	S	S	S	S	S	S	S	S	S	S	S	S	S	S	S	S	S
Erythromycin (15 µg/disk)	S	S	S	S	S	S	S	S	S	S	S	S	S	S	S	S	S	S

S: sensitive, I: Intermediate, R: resistant.

## Supplementary Materials

The following are available online at http://www.mdpi.com/2076-2607/6/3/86/s1, Figure S1: Phylogenetic analysis using 16S rRNA gene by Neighbor-joining method (time-tree).
