# Supplementary material for: Characteristics of Gorilla-Specific Lactobacillus Isolated from Captive and Wild Gorillas
Source: Microorganisms. 2018 Aug 14;6(3):86. doi: 10.3390/microorganisms6030086 (PMC6165273; doi:10.3390/microorganisms6030086)
Supplement: Supplementary file 1 [file microorganisms-06-00086-s001.docx]

**Figure S1.** Phylogenetic analysis using 16S rRNA gene by Neighbor-joining method (time-tree). The time-tree shown was generated using the RelTime method [24]. Divergence times for all branching points in the topology were calculated by the Neighbor-joining methods. Evolutionary analysis were conducted in MEGA version 7.0.20 [24].
